# Integrated Chinese and western medicine for SLE: A protocol for systematic review and meta-analysis

**DOI:** 10.1097/MD.0000000000023597

**Published:** 2021-03-26

**Authors:** Xueli Cheng, Jing Liu, Zhenghong Zhong, Donghan Xu, Xushan Cha, Qibiao Wu

**Affiliations:** a Faculty of Chinese Medicine, State Key Laboratory of Quality Research in Chinese Medicines, Macau University of Science and Technology, Macau; b The First Affiliated Hospital of Guangzhou University of Traditional Chinese Medicine, Guangzhou, Guangdong Province, China.

**Keywords:** Integrated Chinese and Western Medicine, meta-analysis, systematic lupus erythematosus, systematic review

## Abstract

**Background:**

The efficacy and safety of integrated Chinese and western medicine in the treatment of Systemic Lupus Erythematosus (SLE) have not been systematically evaluated.

**Methods:**

This systematic review and meta-analysis will be performed and reported according to Preferred Reporting Items for Systematic Reviews and Meta-Analyses (PRISMA) guidelines. We will search PubMed, Embase, Cochrane Library, Chinese Biomedical Database WangFang, VIP, and China National Knowledge Infrastructure (CNKI) for potentially eligible studies from their inception to Dec. 2020, and we will select all the eligible randomized controlled trials (RCTs) of the integrated Chinese and western medicine for Systemic Lupus Erythematosus. All the studies will be screened according to the inclusion and exclusion criteria and meta-analysis will be conducted using Review Manager V.5.3. and Stata V.12.0. The primary endpoint was the clinical efficacy rate, the secondary outcomes were SLEDAI score, adverse reaction rate, and laboratory test parameters (white blood cells, complement C3).

**Results:**

The results of this study will systematically evaluate the effectiveness and safety of the integrated Chinese and western medicine in the treatment of primary dysmenorrhea.

**Conclusion:**

The efficacy and safety of integrated Chinese and western medicine will be systematically evaluated in this paper, thus providing evidence for the clinical application of this therapy.

**Ethics and dissemination:**

This study is a systematic review, which is based on the published studies, so examination and agreement by the ethics committee are not required in this study. We intend to publish the study results in a journal or conference presentations.

**Open Science Framework (OSF)registration number:**

https://osf.io/mabxh.

## 1 Introduction

Systemic Lupus Erythematosus (SLE) is a chronic autoimmune disease with multisystem damage, and its serum has a variety of antinuclear antibodies.^[1]^ Clinically, SLE can cause organ damage and weak immune function, such as central nervous system damage, renal lesions, etc, and its main clinical manifestations include arthralgia, weight reduction, fever, and fatigue, etc.^[2,3]^ In recent years, a great number of studies have been conducted in the medication and treatment of SLE in modern medicine. In this paper, we will include relevant studies on the clinical efficacy of combination of Chinese traditional and Western medicine in the SLE treatment, and conduct a meta-analysis of the studies meeting the inclusion criteria, hoping to evaluate the clinical efficacy and safety of combination of Chinese traditional and Western medicine in the SLE treatment. The details are as follows.

## 2 Methods

### 2.1 Study registration

The protocol of the systematic review has been registered. Registration: OSF Preregisration. November 3, 2020. https://osf.io/mabxh. This systematic review protocol will be conducted and reported strictly according to Preferred Reporting Items for Systematic Reviews and Meta-Analyses (PRISMA)^[4]^ statement guidelines, and the important protocol amendments will be documented in the full review.

### 2.2 Inclusion and exclusion criteria for study selection

#### 2.2.1 Inclusion criteria

1. The studies included are published randomized controlled trial.^[5]^
2. All the patients were clinically diagnosed as SLE. The treatment group was treated with conventional western medicine (glucocorticoid, namely prednison) combined with decoction of traditional Chinese medicine, with the general course of treatment of 1 to 12 months.

#### 2.2.2 Exclusion criteria

Following studies will be excluded:

(1) Observational study;
(2) Basic study type;
(3) Repeated reports;
(4) Literatures with insufficient data;
(5) Unpublished medical study;
(6) Clinical study with sample size less than 15;
(7) Literatures with insufficient text.^[6]^

### 2.3 Types of participants

All included patients were diagnosed with SLE.

### 2.4 Interventions

The patients in the experimental group were treated with Chinese medicine in addition to routine western medicine (glucocorticoid). The patients in the control group were treated with glucocorticoid.

### 2.5 Outcome measures

The primary endpoint is the clinical efficacy rate, the secondary outcomes are SLEDAI score, adverse reaction rate, and laboratory test parameters (white blood cells, complement C3, etc.).

### 2.6 Search methods

#### 2.6.1 Information sources and search strategies

**Figure 1:**
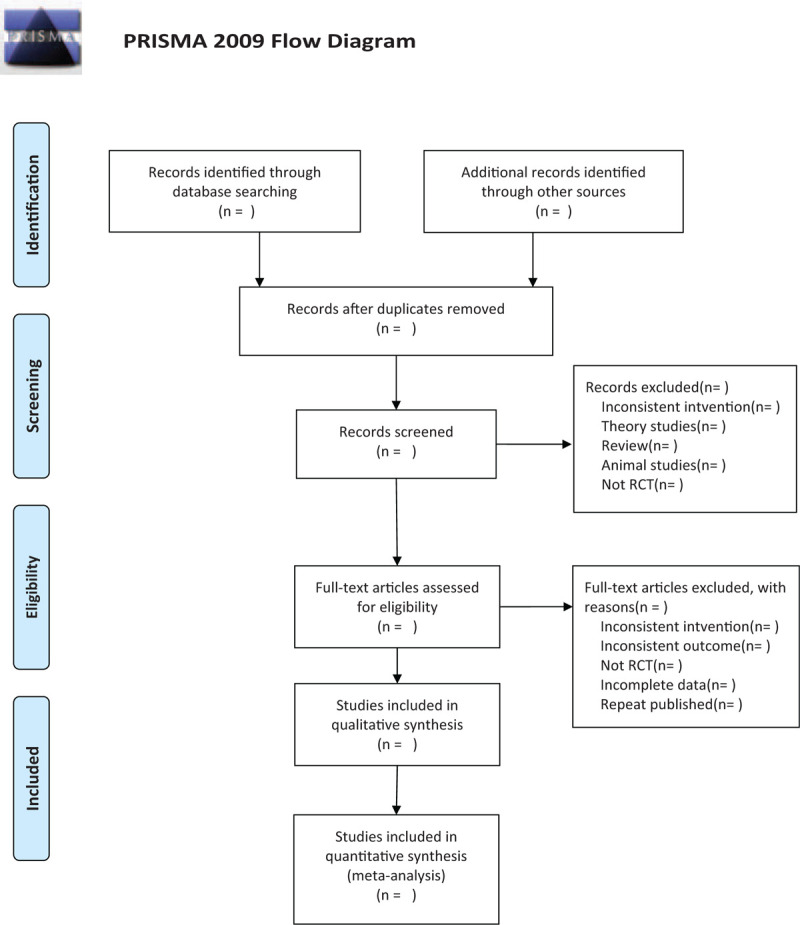
The research flowchart. This figure shows the Identification, Screening, Eligibility, and Included when we are searching articles.

In China National Knowledge Infrastructure (CNKI), VIP, Wanfang, and CBM,^[7]^ Chinese words “integrated Chinese and western medicine ” and “Systemic Lupus Erythematosus” will be used as the key words (Fig. 1), and in the PubMed database provided by the National Center for Biotechnology Information of the United States, the search terms are “Systemic Lupus Erythematosus; Integrated Chinese and Western Medicine; Systematic Review; meta-analysis” (Table 1).

**Table 1:** Search strategy in PubMed database.

#1 randomized controlled trial [Title/Abstract]
#2 controlled clinical trial [Title/Abstract]
#3 randomized [Title/Abstract]
#4 randomly [Title/Abstract]
#5 trial [Title/Abstract]
#6 #1 OR #2 OR #3 OR #4 OR #5
#7 Systematic Lupus Erythematosus[Title/Abstract]
#8 traditional Chinese medicine[Title/Abstract]
#9 Western medicine[Title/Abstract]
#10 #7 OR #8 OR #9
#11 SLE[Title/Abstract]
#12 meta-analysis[Title/Abstract]
#13 Systematic review[Title/Abstract]
#14 #11 OR #12 OR #13
#15 #6 OR #10 OR #14

### 2.7 Data extraction and analysis

#### 2.7.1 Studies selection

Two independent researchers (XL and ZH) will screen all candidate articles based on titles and abstracts according to the inclusion/exclusion criteria, and then retrieve the full text of each potentially relevant trial for further evaluation. Differences of opinion will be resolved by a third independent critic (QB).

#### 2.7.2 Data extraction

Two researchers (XL and ZH) will read all the included text in full, and independently extract the following information:

(1) General information, including trial name and registration information;
(2) Trial characteristic, including trial design, location, setting, and inclusion/exclusion criteria;
(3) The characteristics of the participants, including age, race/ethnicity, course of illness, etc;
(4) Details of intervention, including time of intervention, course of treatment, time of single treatment, etc;
(5) Details of comparison interventions;
(6) Outcomes as described in Section 2.5. If we can not reach an agreement, a third researcher (QB) will make the final decision.

#### 2.7.3 Assessment of risk of bias

The risk of bias in the included RCTs will be independently appraised by 2 researchers (XL and ZH) using the “risk of bias” tool of the Cochrane Collaboration and each included study will be evaluated for quality and risk of bias in accordance with Cochrane manual 5.1.0. In case of any disagreement, a third researcher (QB) will extract the data and results will be attained by consensus. The following criteria will be assessed: random sequence generation; allocation concealment; blinding; incomplete data; selected reporting the results; and other bias. The risk of bias will be classified as “high,” “unclear,” or “low.”

#### 2.7.4 Data analysis

Review Manager 5.3 (Copenhagen: The Nordic Cochrane Centre, The Cochrane Collaboration, 2014) and Comprehensive Meta-Analysis (CMA) 2.0 will be used to combine trials. Continuous outcomes will be expressed as weighted mean difference (WMD) and dichotomous data as risk ratio (RR), with their 95% confidence intervals (CIs). RR is the ratio of the probability of an event occurring in the treatment group to the probability of the event occurring in a control group.

Besides RR, meta-analysis will be also conducted using odds ratio (OR) and risk difference (RD) statistical methods, respectively. Chi-square test and *I*^2^ statistic will be used to measure statistical heterogeneity. When *P* < .05 or *I*^2^ > 50%, substantial heterogeneity will be considered to exist, and the random effects model will be applied to estimate the summary RR (or OR, RD), WMD and 95% CI, otherwise a fixed effects model will be applied. At the same time sensitivity analysis is adopted to determine the robustness of the results.

#### 2.7.5 Patient and public involvement

This is a meta-analysis study based on previously published data, so patient and public involvement will not be included in this study.

#### 2.7.6 Ethics and dissemination

Ethical approval will not be required as this is a protocol for systematic review and meta-analysis. The findings of this study will be disseminated to a peer-reviewed journal and presented at a relevant conference.

#### 2.7.7 Evidence assessed

The quality of evidence for this study will be assessed by “Grades of Recommendations Assessment, Development, and Evaluation (GRADE)” standard.^[8]^ To achieve transparency and simplification, the quality of evidence is divided into 4 levels in GRADE system: high, medium, low, and very low. We will employ GRADE profiler 3.2 for analysis.^[9]^

## 3 Discussion

Glucocorticoids are currently the most common drugs for SLE patients, which can better control the disease activity, and reduce the outbreak of SLE and improve the symptoms of SLE.^[10]^

At the present stage, SLE is clinically treated with western medicine, such as glucocorticoid, immunosuppressants, etc, which cannot achieve the ideal treatment effect, even leads to secondary infection, increases the damage to kidney organs, and even cause death of patients.^[11]^ Although western medicine has a good treatment effect, especially glucocorticoids and immunosuppressants have a positive effect on preventing the pathological lesion of lupus nephritis, there are still serious adverse drug reactions, which have become one of the causes of death of late SLE.

This study shows that the effect of combination of Chinese traditional and western medicine in the SLE treatment is better than that of the single use of glucocorticoids, and the study results have statistical significance. The modern pharmacological study has also proved that traditional Chinese medicine has immune-regulating properties and less side effects. Therefore, The integrated Chinese and western medicine can achive better clinical efficacy in the SLE treatment, provide better treatment for patients, and reduce the side effects, the recurrence rate, and the rebound in the use process of glucocorticoids.

The efficacy and safety of integrated Chinese and western medicine will be systematically evaluated in this study, thus providing evidence for the clinical application of this therapy for SLE.

## Author contributions

**Conceptualization:** Xueli Cheng, Zhenghong Zhong, Jing Liu.

**Data curation:** Donghan Xu.

**Formal analysis:** Xueli Cheng, Zhenghong Zhong.

**Funding acquisition:** Jing Liu, Qibiao Wu.

**Investigation:** Xushan Cha, Jing Liu.

**Methodology:** Xueli Cheng, Zhenghong Zhong, Jing Liu.

**Project administration:** Qibiao Wu.

**Resources:** Xueli Cheng, Zhenghong Zhong, Donghan Xu.

**Software:** Xueli Cheng, Zhenghong Zhong.

**Supervision:** Qibiao Wu, Jing Liu.

**Validation:** Xushan Cha.

**Visualization:** Qibiao Wu.

**Writing – Original Draft:** Xueli Cheng.

**Writing – Review and editing:** Xueli Cheng, Qibiao Wu.
